# Externally controllable glycan presentation on nanoparticle surfaces to modulate lectin recognition†
†Electronic supplementary information (ESI) available: This includes full experimental procedures and additional characterisation data. See DOI: 10.1039/c6nh00202a



**DOI:** 10.1039/c6nh00202a

**Published:** 2016-12-14

**Authors:** Sangho Won, Sarah-Jane Richards, Marc Walker, Matthew I. Gibson

**Affiliations:** a Department of Chemistry , University of Warwick , Coventry , CV4 7AL , UK . Email: m.i.gibson@warwick.ac.uk; b Department of Physics, University of Warwick , Coventry , CV4 7AL , UK; c Warwick Medical School , University of Warwick , Coventry , CV4 7AL , UK

## Abstract

Nature dynamically controls carbohydrate expression on cells rather than static presentation. Here we report synthetic glycosylated nanoparticles that contain polymeric ‘gates’ to enable external control (*via* temperature changes) of glycan surface expression, as an alternative to enzymatic control in nature. This approach offers a new dynamic multivalent scaffold for glycan recognition.

## Abstract 2

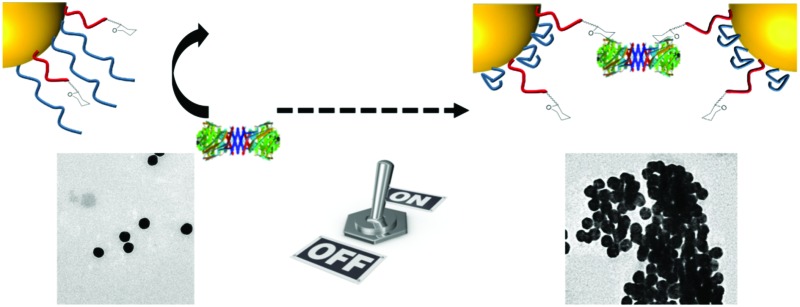
Response polymer gates are employed to enable external control of glycan expression on the surface of multivalent nanoparticles.

Conceptual insights

Carbohydrates dictate an enormous range of processes in biology but are intrinsically complex to study; (i) they are not templated, so genome data does not enable prediction of glycosylation; (ii) glycans on cell surfaces are always changing due to the action of enzymes; they are highly dynamic complicating their study; (iii) current multivalent systems are static and do not reproduce this dynamic presentation This communication presents a new concept to enable dynamic presentation of glycans on a nanoparticle surface as a mimic of the cell surface, using polymers to ‘gate’ access. This is one of the first examples of an (accessible) tool to probe dynamic function of carbohydrates and will be translatable to a range of more complex sugars to probe their function or into advanced biosensors.

Glycans (sugars) mediate a diverse range of biological recognition and signal transduction pathways and are implicated in diseases such as cancer (aberrant glycosylation) or as sites for pathogen adhesion. The ‘readers’ of glycosylation state are lectins; carbohydrate binding proteins which are neither antibodies nor enzymes.^1^ The typical affinity for a glycan to a lectin is rather weak, so Nature presents multiple copies of glycans on cell surfaces to benefit from the cluster glycoside effect – a non-linear enhancement in binding affinity when multiple glycans are present in proximity to each other.^2^ Inspired by this, multivalent systems such as polymers, peptides, surfaces or nanoparticles functionalized with glycans have been used to generate high avidity binders. Due to their high affinity, glycopolymers^3,4^ have been explored as anti-adhesive agents against, HIV,^5^ cholera,^6^ Shiga toxins^7^ and also to recruit growth factors to control stem cell fate^8^ with affinities on the nM scale.

Despite this vast range of structures synthesized, most glycomaterials are static entities with the sugars always accessible for binding. This is in stark contrast to cell-surface glycans which are highly dynamic with the glycans presented changing depending on disease state and for protein folding quality control.^9,10^ Current synthetic materials do not enable control over glycan expression to be modulated, and hence do not fully mimic the natural environment. Dynamic chemical bonds have been used to generate glycopolymers which reconfigure due to the action of lectin binding (*i.e.* internal trigger), but not to an external trigger.^11–13^ In contrast, externally addressable polymers (often termed as ‘smart’ or ‘responsive’) have been extensively studied where an external stimulus, such as light, heat, pH, radiation, metal ions *etc.*, can trigger a (reversible) change in material properties. In particular, thermoresponsive polymers have attracted attention as due to their easy synthesis and diverse range of applications from triggered cellular uptake, trypsin-free cell release^14^ and drug delivery.^15^ Polymers which display an LCST (lower critical solution temperature) undergo a chain collapse (soluble–insoluble) upon heating providing a macroscopic effect from the external trigger.^16,17^ Typical thermoresponsive polymers with an LCST include poly(*N*-isopropylacrylamide) (pNIPAM) and poly[(oligoethylene glycol) methacrylates] (pOEGMAs) due to their transitions being close to 37 °C. Many other classes have been developed and extensively reviewed.^18,19^ Immobilization of responsive polymers onto metal or soft nanoparticle enables dynamic control over aggregation state based on an external trigger.^20,21^ Mastrotto *et al.* used pNIPAM collapse to expose folate moieties on gold nanoparticle surfaces to enable temperature triggered uptake into cancerous cell lines.^22^ Temperature gating has also been used to control access to biotin functionality on glass surfaces.^23^ Gold nanoparticles (AuNPs) are widely used due to their easy functionalization with thiols and unique optical properties which make them excellent contrast agents in electron microscopy, or dark field microscopy, but also as colorimetric sensors due to the coupling of their SPR bands when aggregated leading to a red-blue colour shift.^24^ Immobilization of glycans onto AuNPs has been used as biosensors. Field *et al.*, used α2,6-thio-linked sialic acid to detect human influenza,^25^ and Richards *et al.* have used glycosylated gold nanoparticles libraries as multiplex sensors.^26^

Considering the above, we reasoned that if a nanoparticle surface could be formulated correctly, a responsive polymer could be used as an externally addressable ‘gate’ which upon application of a stimulus, is ‘opened’ (*via* chain collapse) to enable access to a glycan and hence enable binding. This can be considered as a synthetic alternative to enzyme expression levels, which *in vivo* control glycan expression based upon biological triggers.

To provide the desired gating mechanism on the nanoparticle surface pNIPAM was selected as the thermo-responsive polymer due to its well characterized switchable behavior and high grafting density onto gold.^27^ Well-defined poly(hydroxyethyl acrylamide) (pHEA) was selected as a non-responsive co-coating as we have previously demonstrated it to be an excellent stabilizing polymer for glyco-nanoparticles.^28^ RAFT (reversible activation fragmentation transfer) polymerization^29^ was employed as it enables control over molecular weight and also installs sulfur containing end-groups for direct conjugation onto AuNPs.^29^ pHEA was synthesized using a pentafluorophenyl ester RAFT agent, which enabled quantitative installation of 2-deoxy, 2-amino galactose post-polymerization, confirmed by ^19^F NMR, Fig. 1A. Well-defined pHEA with DP = 15 was obtained, along with two different pNIPAMs of DP 25 or 50, confirmed by size exclusion chromatography and NMR, Table 1. As expected, the pNIPAMs displayed a lower critical solution temperature in solution (Table 1 and ESI†) of approximately 36 °C, which is essential for the gating concept, (below). All polymers were purified by dialysis against water prior to use.

**Fig. 1:**
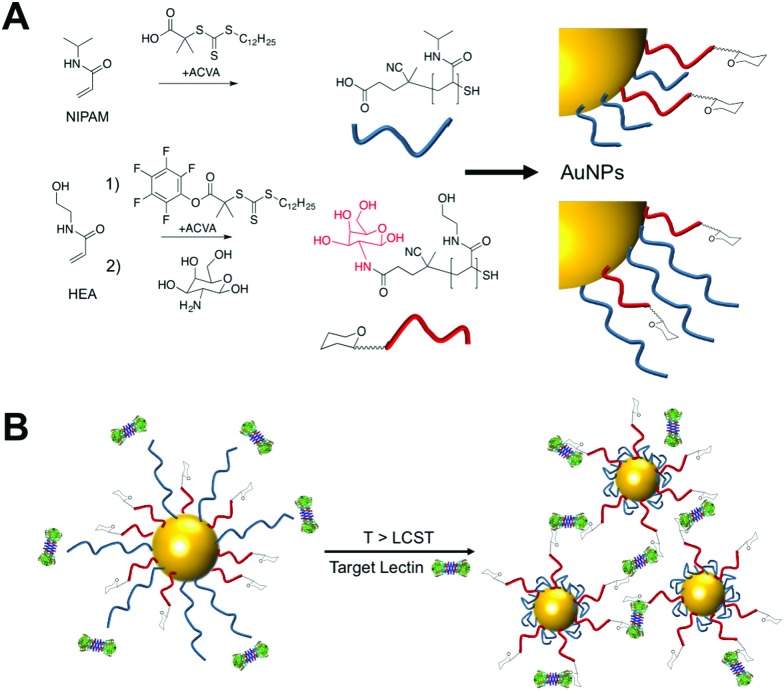
(A) Synthesis of polymers by RAFT. (B) Concept of using responsive polymers to gate access to nanoparticles. Below the LCST of pNIPAM steric hindrance prevents lectin binding to glycans, but above the LCST, the polymer collapse to expose glycans enabling binding and aggregation of the particles.

**Table 1:** Polymers used in this study

Polymer	[M]/[CTA]/[I] [mol]	*M*_n_ target [g mol^–1^]	Conversion^*a*^ [%]	*M*_n Theo_ ^*b*^ [g mol^–1^]	*M*_n SEC_ ^*c*^ [g mol^–1^]	*M*_w_/*M*_n_ [–]	Cloud point^*d*^ [°C]
pNIPAM_25_	25/1/0.2	3200	87%	2800	2900	1.07	36
pNIPAM_50_	50/1/0.2	6000	86%	5200	7100	1.10	38
PFP–pHEA_15_	15/1/0.2	2300	93%	2100	4800	1.10	—

^*a*^Determined ^1^H NMR.

^*b*^Calculated from the [monomer] : [CTA] ratio and of conversion.

^*c*^Determined by SEC in DMF using PMMA standards.

^*d*^Cloud point was measured in water upon heating from 25 °C to 80 °C, 1.0 mg mL^–1^ polymer concentration.

The gating concept, whereby increasing the temperature leads to selective collapse of the pNIPAM chains and consequently exposing the glycan is shown in Fig. 1B. The polymer coatings had to fulfil several criteria for this concept to be successful (i) long enough to promote colloidal stability; (ii) correct ratio of responsive and glycan bearing polymer to ensure multivalent enhancement, but retain colloidal stability after pNIPAM collapse; (iii) provide enough steric bulk to block glycan access below collapse temperature. Initial screens using pHEA_15_-Gal coated nanoparticles in lectin aggregation assays (ESI†) indicated that 60 nm nanoparticles gave larger and faster responses than 40 nm particles and hence is the diameter used from this point onwards (ESI†).^28^ XPS (X-ray photo electron spectroscopy) was used to provide chemical evidence of the grafting-to success. No significant differences in density (but exact values could not be obtained) were seen between the polymers used on their own, suggesting that the feed ratio will be close to the obtained ratio on the particle surface. Assuming 0.3 chains nm^–2^, this gives ∼3000 chains (and glycans) per particle.

In a first series of experiments pHEA_15_-Gal was mixed with either pNIPAM_25_ or pNIPAM_50_ in a mass ratio of 8 : 2 and used to functionalize 60 nm AuNPs. These particles were evaluated for binding with SBA (Soy Bean Agglutinin) which has a preference for GalNAc binding and would hence lead to aggregation of GlycoAuNPs. With the pNIPAM_25_ coating, addition of SBA at either 20 or 40 °C (*i.e.* above and below the LCST of pNIPAM) a clear change in colour was observed from red to blue, with a shift in the Abs_max_ to longer wavelengths. This indicated that the pNIPAM_25_ provided an insufficient steric block and that the Gal-residues were accessible at both temperatures. Switching to the pNIPAM_50_ (same ratio) coating, and again exposing to SBA showed no interaction with SBA after 30 minutes incubation at 20 °C showing that this polymer was sufficiently bulky to limit access to the Gal residues. Upon increasing the temperature to 40 °C, in the presence of SBA, there was a small, but significant shift in the UV-Vis spectra with an increase at 700 nm and decrease at 540 nm indicative of lectin binding and aggregation. This clearly demonstrated that the concept of responsive gating to glycan access could be achieved, but that the surface coating has to be precisely tuned to achieve the balance required (Fig. 2).

**Fig. 2:**
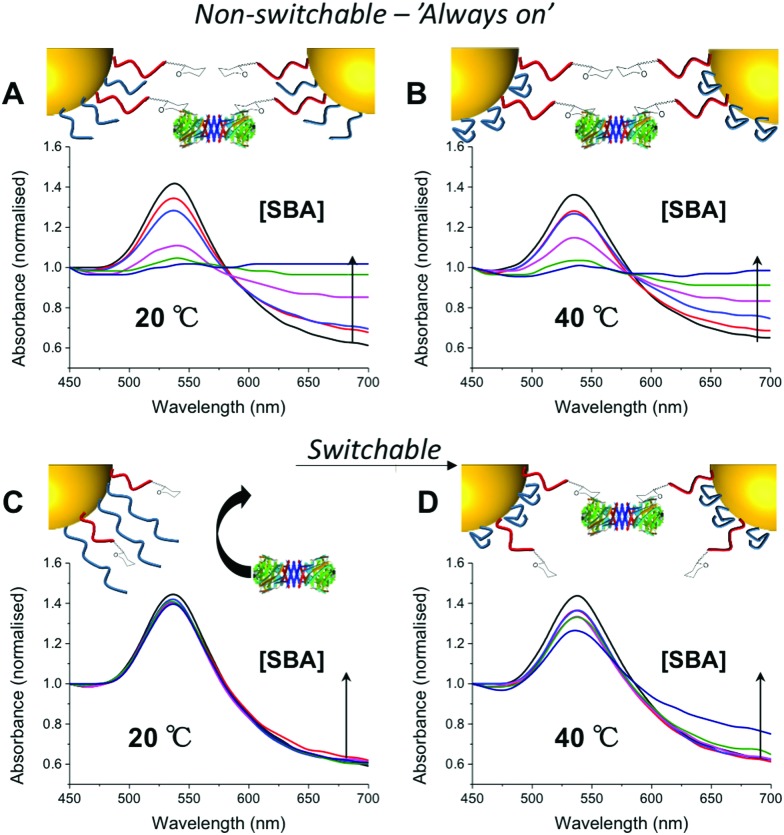
UV-Vis traces of different nanoparticle formulations in presence of serial dilution of SBA (10–1 μg mL^–1^) after 30 minutes incubation. All particles (60 nm) had [pHEA_15_-Gal] : [pNIPAM_x_] 8 : 2. pNIPAM_25_ at 20 °C (A) and 40 °C (B); pNIPAM_50_ at 20 °C (C) and 40 °C (D). An increase in Abs_700_ and decrease in Abs_540_ is indicative of binding. All curves normalise so Abs_450_ = 1.

Further optimization studies revealed that changing the ratio of pHEA_15_-Gal : pNIPAM_50_ from 8 : 2 to 9 : 1 provided the optimum balance between glycan affinity (*i.e.* aggregation) and switchability (ESI†). To ensure the changes seen were due to particle aggregation (indicative of lectin-cross-linking), SBA binding was investigated using dynamic light scattering and transmission electron microscopy (TEM) Fig. 3. The optimized nanoparticle formulation (above) was incubated with SBA at both 20 and 40 °C for 30 minutes and the observed hydrodynamic diameters shown. At 20 °C, in the presence of various concentrations of SBA there was no change in hydrodynamic diameter from the initial 60 nm. At 40 °C, with no SBA added, the particles were stable with the initial diameter of ∼80 nm (due to some surface reconfiguration compared to at 40 °C) being retained, in agreement with the UV-Vis data. Following 30 minutes of incubation there was a clear dose-dependent increase in the aggregate size as [SBA] was increased, again supporting the hypothesis that the pNIPAM is gating access to the glycan. TEM analysis was also conducted to provide direct evidence of temperature triggered lectin/particle agglutination. Fig. 3C shows nanoparticles plus SBA at 20 °C, which are clearly well-dispersed and Fig. 3D shows the aggregates which form upon heating to 40 °C only in the presence of SBA (Fig. 4).

**Fig. 3:**
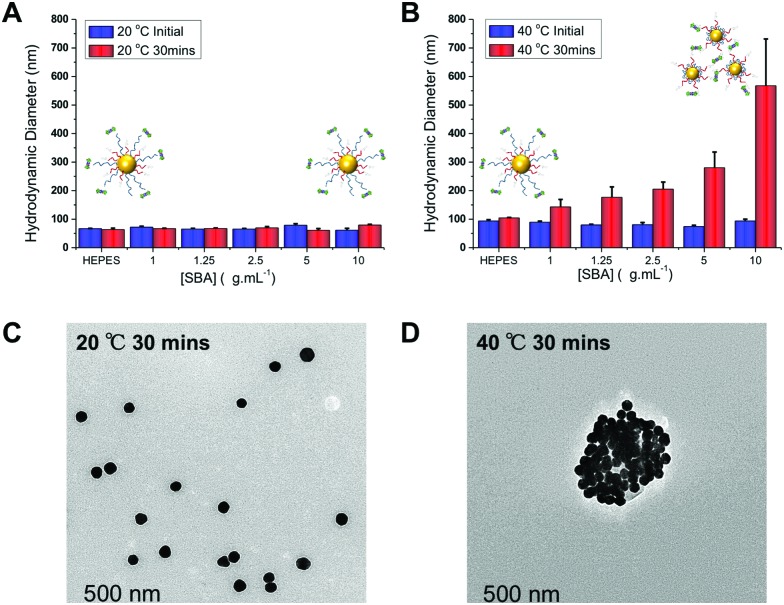
Dynamic light scattering (DLS) analysis of thermally gated lectin binding. pHEA_15_-Gal : pNIPAM_50_ ratio 9 : 1 @AuNP_60_. (A) Hydrodynamic diameter at 20 °C initially, and after 30 minutes incubation with SBA; (B) hydrodynamic diameter at 40 °C, initially and after 30 minutes incubation with SBA. All results are mean from a minimum of 3 independent repeats. TEM images of these particles after addition of SBA (10 μg mL^–1^): (C) at 20 °C for 30 min; (D) at 40 °C following 30 minutes incubation.

**Fig. 4:**
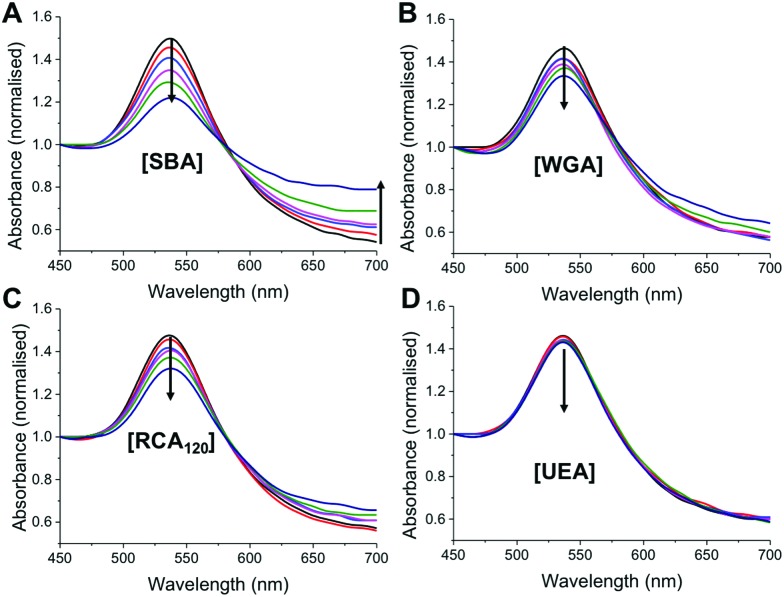
Assessment of specificity of thermally gated nanoparticles to a panel of lectins. Lectin concentration is 1–10 μg mL^–1^. (A–D) Show UV-Visible traces (normalised to Abs_450_) following 30 minutes incubation at 40 °C with indicated lectin. Arrow indicates increase concentration.

As a final test of the system, the optimized nanoparticle formulation (with the 9 : 1 ratio of pHEA_15_-Gal : pNIPAM_50_) was interrogated with a panel of lectins, with different binding specificities^30^ at both 20 °C and 40 °C. SBA, WGA (Wheat germ agglutinin), UEA (*Ulex europaeus* agglutinin) and RCA120 (*Ricinus communis* agglutinin) were employed. At 20 °C there was no measurable change in UV-Vis spectra upon incubation with SBA, WGA, UEA or RCA_120_ lectins, indicating that the glycan is sterically shielded against all the lectins (ESI†). Increasing the temperature to 40 °C, however, lead to clear changes in the UV-Vis spectra for SBA, RCA_120_ and WGA as would be expected with their known affinities for Gal (or GalNAc/GluNAc for WGA). The control lectin UEA, which has specificity for fucose residues did not bind at any temperature, proving the specificity of the interaction and that temperature-induced aggregation is not a factor. A partial isotherm showing the relative changes is included in the ESI† as a measure of the relative affinity, in the order SBA > WGA ∼ RCA_120_ ⋙ UEA. Additional control experiments using BSA as a model non-carbohydrate binding protein revealed there were not non-specific interactions (see ESI†). The ability to control glycan expression would be a powerful tool for studying the role of multivalency intracellularly, where the glycan is only exposed one trafficked to the desired location, potentially provided spatiotemporal control. They could also be used as new biomolecular logic gates.

In summary, we have demonstrated a new concept in glyco-engineering where responsive polymer surfaces, rather than external enzyme expression levels, control the display of sugars on the surface of a nanoparticle, which could be considered a simple cell mimetic. The ‘gate’ pNIPAM had to be added in a relatively low ratio compared to the glycan-bearing polymer to ensure a binary on/off effect, with significant lectin binding above the pNIPAM LCST observed. The specificity of the GlycoAuNP was confirmed against a panel of lectins, which glycan expression only being induced above the critical temperature. The complex function of this relatively simple system with in-built optical outputs (AuNP colour changes) is highly versatile and presents a new method to dynamically control glycan expression using fully synthetic systems. By controlling presentation on the AuNP surface with an external trigger, we can envisage this being used as a tool to probe glycan function under very controlled environments, including intracellularly and could be considered a molecular ‘AND’ gate. Furthermore, the pNIPAM collapse could be replaced with a range of other stimuli responsive polymers, to enable biochemical rather than temperature trigger to probe more complex cellular environments.

MIG acknowledges the ERC for a Starting Grant, CRYOMAT 638661 and BBSRC (BB/M02878X/1). SW thanks UoW for a chancellors international scholarship. SJR was a UoW IAS Early Career fellow.
