# Supplementary material for: Externally controllable glycan presentation on nanoparticle surfaces to modulate lectin recognition
Source: Nanoscale Horiz. 2016 Dec 14;2(2):106–9. doi: 10.1039/c6nh00202a (PMC5625543; doi:10.1039/c6nh00202a)
Supplement: Supplementary file 1 [file NH-002-C6NH00202A-s001.pdf]

Electronic Supplementary Material (ESI) for Nanoscale Horizons.
This journal is © The Royal Society of Chemistry 2016

Supporting Information

Externally controllable glycan presentation on nanoparticle surfaces to modulate lectin recognition

Sangho Won,[a] *Sarah-Jane Richards*[a] *and Matthew I. Gibson*[a,b]*

[a] Department of Chemistry, University of Warwick, Gibbet Hill Road, Coventry, CV47AL, UK

[b] Warwick Medical School, University of Warwick, Gibbet Hill Road, Coventry, CV47Al, UK

Corresponding Author; M.i.gibson@warwick.ac.uk

Experimental Section

Materials

All chemicals were used as supplied unless otherwise stated. Methanol, hexane, hydrochloric acid, dichloromethane, toluene, acetone, tetrahydrofuran and diethyl ether were purchased from Fisher Scientific at laboratory reagent grade. Deuterochloroform (99.9 atom % D), deuteromethanol (99.5 atom % D), 4,4'-azobis(4-cyanovaleric acid) (> 97.0 %), dodecane thiol (≥ 98.0 %), potassium phosphate tribasic (reagent grade, ≥ 98.0 %), carbon disulfide (≥ 99.9 %), 2-bromo-2-methylpropionic acid (98.0 %), *N*-isopropylacrylamide (97.0 %), hydroxyethylacrylamide (97.0 %), *N*-(3-dimethylaminopropyl)-*N*'-ethylcarbodiimide hydrochloride (98.0 %), mesitylene (analytical standard) and magnesium sulfate (≥ 99.5 %) were all purchased from Sigma-Aldrich. 4-(Dimethylamino)pyridine (99.0 %), pentafluorophenol (99.0 %) and trimethylamine (99.0 %) were purchased from Acros. Clear, polystyrene, flat-bottom, half-area 96-well microtiter plates were purchased from Greiner Bio-one. 10 mmol HEPES buffer containing 0.15 M NaCl, 0.1 mM $CaCl_2$ and 0.01 mM $MnCl_2$ (pH 7.5, HEPES) was prepared in 200 mL of milliQ water (with a resistance of 18.2 MΩ cm). Gold nanoparticle solutions for 40 nm (0.296 mmol L^{-1}) and 60nm (0.288 mmol L^{-1}) were purchased from BBI Solutions. Soybean agglutinin, Ricinus communis agglutinin, Ulex Europaeus Agglutinin and Wheat Germ Agglutinin were purchased from Vector Labs. D-Galactosamine hydrochloride was purchased from Carbosynth Ltd.

Physical and analytical methods

^{1}H, ^{13}C and ^{19}F NMR spectra were recorded for analysis of monomer conversions and polymer compositions on Bruker HD-300 and HD-500 spectrometer. All chemical shifts are reported in ppm (δ) relative residual non-deuterated solvent. FTIR spectra were acquired using a Bruker

Vector 22 FTIR spectrometer with a Golden Gate diamond attenuated total reflection cell. A total 64 (or 128) scans with resolution of 4 cm^{-1} were collected. Samples were pre-dried as a thin film for FTIR analysis. SEC analysis was conducted on Varian 390-LC MDS system equipped with a column, two PL-AS RT/MT auto sampler, a PL-gel 3 mm (50 × 7.5 mm) guard column, two PL-gel 5 mm (300 × 7.5 mm) mixed-D columns using dimethylformamide (DMF) with 1 mg mL^{-1} LiBr at 50 °C as the eluent at a flow rate of 1.0 mL min^{-1}. The GPC system was equipped with ultraviolet (UV) (set at 280 nm) and differential refractive index (DRI) detections. Narrow molecular weight poly(methyl methacrylate) (PMMA) standards (200-1.0 × 106 g mol^{-1}) were used for calibration using a second order polynomial fit. Polymer solutions at 1 mg mL^{-1} were prepared in the eluent and filtered through 0.45 mm filters prior to injection. UV-vis spectra were recorded in a disposable cuvette using a Cary 60 UV-vis spectrometer from Agilent at 25 °C. Lower critical solution temperatures of free pNIPAM and pNIPAM nanoparticles were also analyzed using an Agilent Cary 60 UV-vis spectrometer equipped with a temperature controller at 700 nm with a heating/cooling rate of 1 °C min^{-1}. The cloud point of pNIPAM and pNIPAM nanoparticles were determined by normalising the turbidimetry curve such that the values were in the range of 0 to 1, and the transition temperature was defined as being the temperature corresponding to a normalised absorbance of 0.5. A polymer concentration of 1.0 mg mL^{-1} was used in all experiments. DLS measurements were performed using a Nano-Zs from Malvern Instruments, UK running DTS software (4 mW, He-Ne laser, λ = 633 nm) and an avalanche photodiode (APD) detector. The scattered light was measured at an angle of 173° for DLS measurement. The temperature was stabilized to ± 0.1 °C of the set temperature. All samples were prepared at the concentration of 0.057 mg mL^{-1} gold nanoparticles. Hydrodynamic radii were determined using the manufacturer's software. Absorbance measurements of the nanoparticles incubated with lectin were recorded on a BioTek SynergyTM HT multi-detection microplate reader obtained using Gen5 1.11 multiple

data collection and analysis software. The size and morphology of the synthesized gold nanoparticles and polymer coated gold nanoparticles were estimated by JEOL 2100FX transmission electron microscopy (TEM) at an accelerating voltage 200 kV. A drop of sample solution was deposited onto a copper grid and the water was evaporated under air. No staining was applied. The x-ray photoemission spectroscopy (XPS) data were collected at the Warwick Photoemission Facility, University of Warwick. The samples investigated in this study were deposited on to Cu foil, mounted on to a sample bar and loaded in to a Kratos Axis Ultra DLD spectrometer which possesses a base pressure of ~ 5×10^{-10} mbar. XPS measurements were performed in the main analysis chamber, with the sample being illuminated using an Al kα x-ray source. The measurements were conducted at room temperature and at a take-off angle of (30°) with respect to the surface parallel. The core level spectra were recorded using a pass energy of 20 eV (resolution approx. 0.4 eV). The spectrometer work function and binding energy scale were calibrated using the Fermi edge and $3d_{5/2}$ peak recorded from a polycrystalline Ag sample immediately prior to the commencement of the experiments. The data were analysed in the CasaXPS package, using Shirley backgrounds, mixed Gaussian-Lorentzian (Voigt) lineshapes. For compositional analysis, the analyser transmission function has been determined using Ag, Au and Cu foils to determine the detection efficiency across the full binding energy range.

Experimental procedures

Synthesis of 2-(dodecylthiocarbonothioylthio)-2-methylpropanoic acid (DMP)

Dodecane thiol (4.00 g, 4.73 mL, 19.76 mmol) was added dropwise to a stirred suspension of K_3PO_4 (4.20 g, 19.76 mmol) in acetone (60 mL) over 25 minutes. CS_2 (4.10 g, 3.24 mL, 53.85 mmol) was added and the solution turned bright yellow. After stirring for ten minutes 2-bromo-2-methylpropionic acid (3.00 g, 17.96 mmol) was added and a precipitation of KBr was noted. After stirring for 16 hour, the solvent was removed under reduced pressure and the residue was extracted into CH_2Cl_2 (2 × 200 mL) from 1M HCl (200 mL). The organic extracts were washed with water (200 mL) and brine (200 mL) and further dried over $MgSO_4$. The solvent was removed under reduced pressure and the residue was purified by recrystallization in hexane.

^{1}H NMR (300 MHz, $CDCl_3$) δ_{ppm}: 3.31 (2H, t, $J_{12\text{-}11}$= 7.34 Hz, H^{12}); 1.76 (6H, s, H^{13}); 1.70 (2H, m, H^{11}); 1.41 (2H, m, H^{10}); 1.28 (16H, br. s, $H^{2\text{-}9}$); 0.90 (3H, t, $J_{1\text{-}2}$= 6.79 Hz, H^1).

^{13}C NMR (500 MHz, $CDCl_3$) δ_{ppm}: 220.98 (C^{13}); 176.63 (C^{16}); 55.44 (C^{14}); 37.09 (C^{12}); 31.92, 29.64, 29.56, 29.45, 29.35, 29.24, 29.11, 22.70 ($C^{2\text{-}9}$); 28.96 (C^{10}); 27.81 (C^{11}); 25.27 (C^{15}); 14.13 (C^1).

IR cm^{-1}: 2916 (alkyl-H stretch); 1710 (C=O stretch); 1068 (S-(C=S)-S stretch).

Polymerisation of N-isopropylacrylamide using 2-(dodecylthiocarbonothioylthio)-2-methylpropanoic acid (DMP*)*

Polymers with three different molecular weights were synthesised in typical procedure.[33] N-isopropylacrylamide (1 g, 8.84 mmol), 2-(dodecylthiocarbonothioylthio)-2-methylpropanoic acid (64.45 mg, 177 μmol), and 4,4'-azobis(4-cyanovaleric acid) (ACVA) (9.9 mg, 35.3 μmol) were dissolved in methanol/toluene (1 : 1; 4mL) in a glass vial containing a stir bar giving [monomer] : [chain transfer agent] : [initiator] = 50 : 1 : 0.2. Mesitylene (150 μL) was added

as an internal reference and the mixture was stirred (5 mins). An aliquot of this starting mixture was removed for ^{1}H NMR analysis. The vial was fitted with a rubber septum and degassed by bubbling with nitrogen gas (30 mins). The vial was then placed in an oil bath thermostated at 70 °C. After 35 minutes, the reaction mixture was opened to air and quenched in liquid nitrogen. An aliquot was removed and conversion determined by ^{1}H NMR. The remainder was precipitated into diethyl ether (45 mL). The polymer was re-precipitated and purified from THF to diethyl ether three times. The product was purified three times by precipitation from toluene into diethyl ether, isolated centrifugation, and dried under vacuum overnight to give a yellow solid. The overall monomer conversion was determined from the ^{1}H NMR spectrum by measuring the decrease in intensity of the vinyl peaks associated with the monomer relative to mesitylene. Conversion (NMR): 86 %; M_n (theoretical), 5200 g mol^{-1}; M_n (SEC), 7100 g mol^{-1}; M_w/M_n (SEC), 1.10.

Synthesis of pentafluorophenyl 2-(dodecylthiocarbonothioylthio)-2-methylpropanoic acid (PFP-DMP)

2-(dodecylthiocarbonothiolythio)-2-methylpropanoic acid (0.50 g, 1.37 mmol), N-(3-dimethylaminopropyl)-N'-ethylcarbodiimide hydrochloride (EDC) (0.39 g, 2.05 mmol) and 4-(dimethylamino)pyridine (DMAP) (0.25 g, 2.05 mmol) in 40 mL dichloromethane (DCM) was stirred for 20 minutes under N_2. Pentafluorophenol (0.78 g, 4.24 mmol) in 5 mL DCM was added. The reaction was stirred overnight at room temperature. The reaction was washed successively with 3 M HCl (50 mL), 1 M $NaHCO_3$ (50 mL) and 0.5 M NaCl (50 mL). The reaction was then dried over $MgSO_4$, filtered and then concentrated in vacuum.

^{1}H NMR (300 MHz, $CDCl_3$) δ_{ppm}: 3.24 (2H, t, $J_{12\text{-}11}$= 7.70 Hz, H^{12}); 1.79 (6H, s, H^{13}); 1.65 (2H, m, H^{11}); 1.32 (2H, m, H^{10}); 1.18 (16H, br. s, $H^{2\text{-}9}$); 0.81 (3H, t, $J_{1\text{-}2}$= 6.42 Hz, H^{1}).

^{13}C NMR (500 MHz, $CDCl_3$) δ_{ppm}: 219.94 (C^{13}); 169.62 (C^{16}); 55.41 (C^{14}); 37.17 (C^{12}); 31.92, 29.63, 29.55, 29.43, 29.35, 29.26, 29.09, 22.70 (C^{2-9}); 28.92 (C^{10}); 27.82 (C^{11}); 25.43 (C^{15}); 14.12 (C^{1}).

^{19}F NMR (300 MHz, $CDCl_3$) δ_{ppm}: -151.5 (d, 2F, ortho F); -157.7 (t, F, para F); -162.4 (t, 2F, meta F).

IR cm^{-1}: 2923 (CH_2); 1779 (C_6F_5C=O); 1073 (S-(C=S)-S).

Polymerisation of hydroxyehtylacrylamide usting pentafluorophenyl 2-(dodecylthiocarbonothioylthio)-2-methylpropanoic acid (PFP-DMP)

In a typical reacation, hydroxyehtylacrylamide (HEA) (0.50 g, 4.34 mmol), pentafluorophenyl 2-(dodecylthiocarbonothiolythio)-2-methylpropanoic acid (PFP-DMP) (0.154 g, 0.289 mmol), 4,4'-azobis(4-cyanovaleric acid) (ACVA) (0.0162 g, 0.058 mmol) were dissolved in 50 : 50 toluene : methanol (4 mL). Mesitylene (150 μL) was added as an internal reference. An aliquot was taken for NMR analysis in MeOD. The solution was degassed under N_2 for 30 mins. The reaction was stirred at 70 °C for 90 mins. An aliquot was taken for NMR analysis for NMR analysis in MeOD. The reaction was rapidly cooled in liquid nitrogen and precipitaed into diethyl ether. The polymer was reprecipitaed into diethyl ether from methanol twice to yield a yellow polymer product which was dried under vacuum. Conversion (NMR): 93 %; M_n (theoretical), 2100 g mol^{-1}; M_n (SEC), 4800 g mol^{-1}; M_w/M_n (SEC), 1.10.

End group modification of PFP-polyhydroxyethylacrylamide using galactosamine

In a typical reacation, PFP-pHEA (50 mg, 0.024 mmol), galactosamine (25.2 mg, 0.117 mmol) were dissolved in 5 mL DMF with 0.05 M triethylamine (TEA). The reaction was stirred at 50 °C for 16 hrs. The polymer was precipitated into diethyl ether from methanol three times and dried under vacuum. IR indicated loss of C=O stretch corresponding to the PFP ester.

General procedure for the synthesis of polymer-coated gold nanoparticles

Approximately 1 mg of the desired thiol-terminated polymer (pNIPAM or pHEA- Gal) was added to a microcentrifuge tube, and dissolved in 100 μL of high-purity water. 900 μL of the citrated-stabilized gold nanoparticle solution was added to this tube (40 nm: 0.296 mmol L^{-1}, 60 nm: 0.288 mmol L^{-1} total gold concentration), which was then agitated 30 mins in the absence of light. To remove excess polymer, the particles were centrifuged and following careful decantation of the supernatant, the particles were then re-dispersed in 1 mL of high-quality water and the centrifugation-resuspension process repeated for a total of 3 cycles. After the final cycle the particles were dispersed in 1 mL of high-quality water for future use. Assuming complete incorporation of the citrate coated gold particles into the final polymer coated particles the total concentration of gold in the final solution was 0.296 mmol L^{-1}, 0.058 mg mL^{-1} and 0.288 mmol L^{-1}, 0.057 mg mL^{-1}.

Gold nanoparticle functionalisation using a mixture of pNIPAM and pHEA-Gal (1 : 9 molar ratio)

100 μL of total polymer solution with different molar ratio between DP50 pNIPAM (0.25 mg, 4.76 mmol,

10 μL) and pHEA-Gal (0.90 mg, 4.76 mmol, 90 μL) was added to 900 μL of 60 nm gold nanoparticles. Left for 30 minutes at room temperature and centrifuged to remove any attached polymer and resuspended in water.

Lectin induced aggregation studies by absorbance

A stock solution of the lectin was made up (0.1 mg mL^{-1} for SBA) in 10 mM HEPES buffer with 0.15 M NaCl, 0.1 mM $CaCl_2$ and 0.01 mM $MnCl_2$. 25 μL serial dilution was made up in the same buffer in a 96-well micro-titre plate. 25 μL of the multi-polymer functionalised gold

nanoparticle were added to each well. Initial and after 30 minutes, both absorbance spectrum were recorded from 450 nm – 700 nm with 10 nm intervals at 20 °C and 40 °C, respectively.

Additional Data

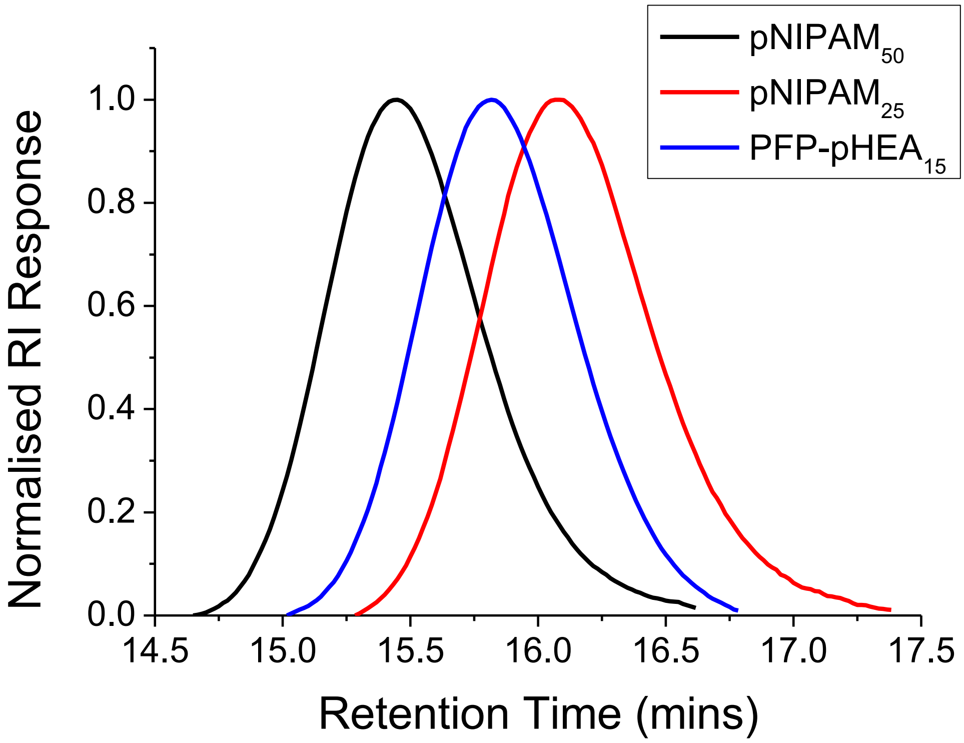

Figure S1. SEC analysis of polymers

Infrared analysis of PHEA before and after reaction of pentafluorophenol end-group with galactosamine. Disappearance of C=O at around 1750 cm-1 attributable to the carbonyl associated with the PFP end-group being removed.

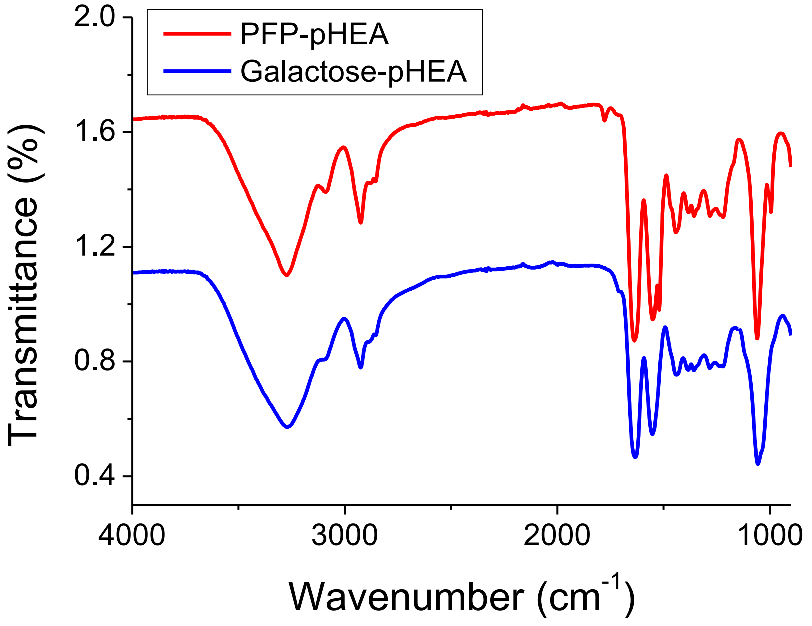

Figure S2. Infrared spectra of PFP-pHEA (red) and galactosamine-pHEA (blue) showing removal of PFP group.

Table S1. Gold nanoparticles with pNIPAM and pHEA-Gal functionalisation.

Particle	SPR uncoated [nm][a)]	SPR polymer coated [nm][b)]	Diameter [nm][c)]	Cloud point HEPES [°C][d)]
**Bare gold 40 nm**	527	-	42	-
**$pHEA_{15}$-Gal@Au40**	527	530	50	-
**Bare gold 60 nm**	534	-	58	-
**$pHEA_{15}$-Gal@Au60**	534	537	66	-
**$pNIPAM_{25}$@Au60**	534	537	64	64
**$pNIPAM_{25}$(8)/$pHEA_{15}$-Gal(2)@Au_{60}**	534	537	64	67
**$pNIPAM_{25}$(5)/$pHEA_{15}$-Gal(5)@Au_{60}**	534	537	66	72
**$pNIPAM_{25}$(2)/$pHEA_{15}$-Gal(8)@Au_{60}**	534	538	66	78
**$pNIPAM_{50}$@Au60**	534	538	75	61
**$pNIPAM_{50}$(2)/$pHEA_{15}$-Gal(8)@Au_{60}**	534	538	68	50
**$PNIPAM_{50}$(1)/$pHEA_{15}$-Gal(9)@Au_{60}**	534	538	67	52

[a)]SPR maximum of 60 nm of uncoated gold nanoparticles; [b)]SPR maximum of gold nanoparticles after coating with polymer; [c)]Z-average diameter determined by DLS; [d)]Cloud point was measured in HEPES buffer upon heating from 25 °C to 80 °C, 0.057 mg mL^{-1} total particle concentration. The CP (cloud point) of particles defined as being the point of 50% transmittance by UV-Vis spectroscopy.

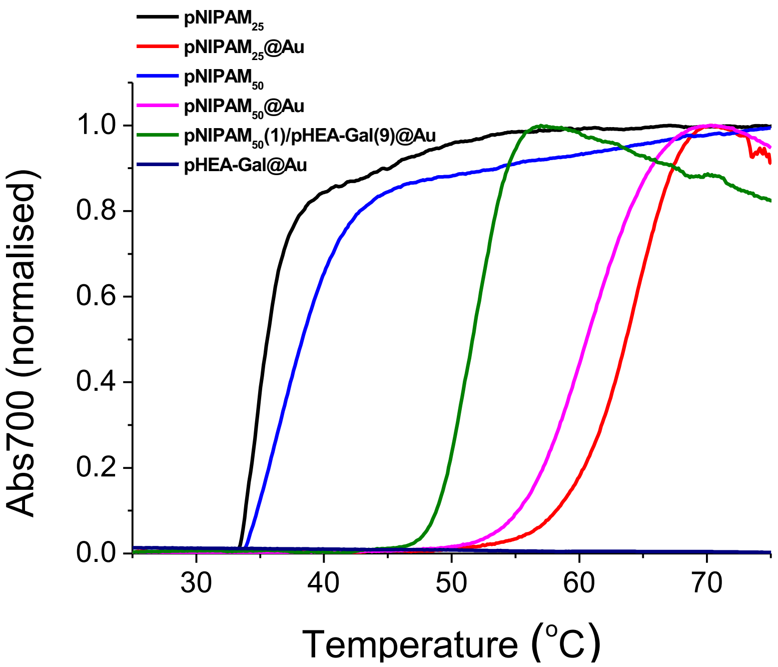

Figure S3. Turbidimetry scans (absorbance at 700 nm) of pure pNIPAM (1.0 mg mL^{-1}) and polymer functionalised gold nanoparticles (0.057 mg mL^{-1}).

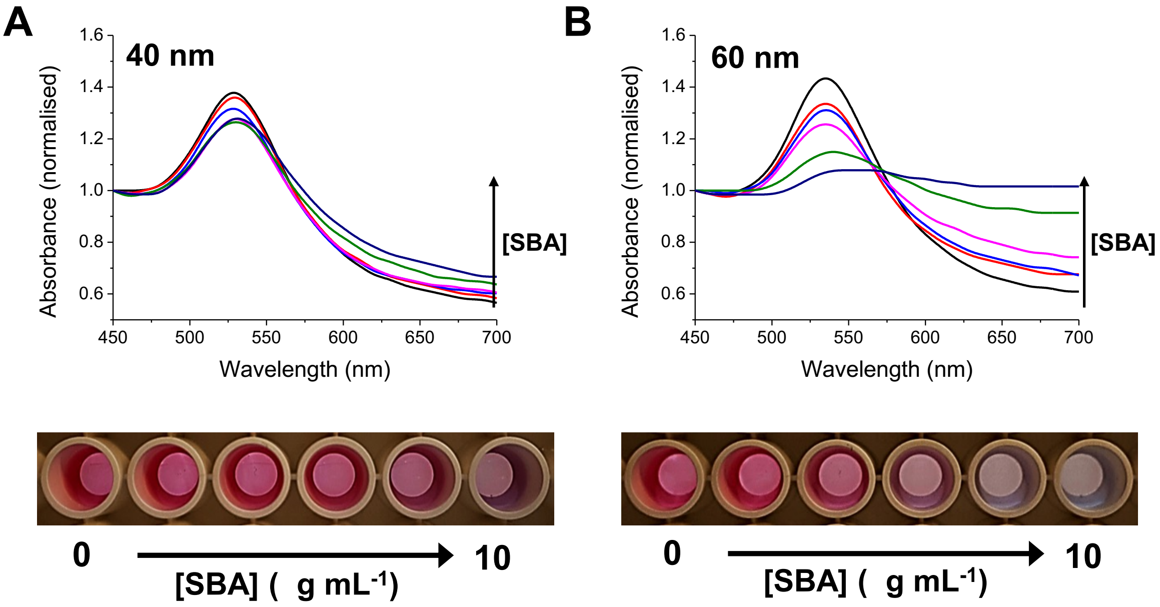

Figure S4. UV-vis spectrum and photographs of colour changes of pHEA$_{15}$-Gal coated A) 40 nm and B) 60 nm sized gold nanoparticles upon addition of serial dilution of SBA (0 – 10 μg mL^{-1}) following 30 mins of incubation at 20 °C.

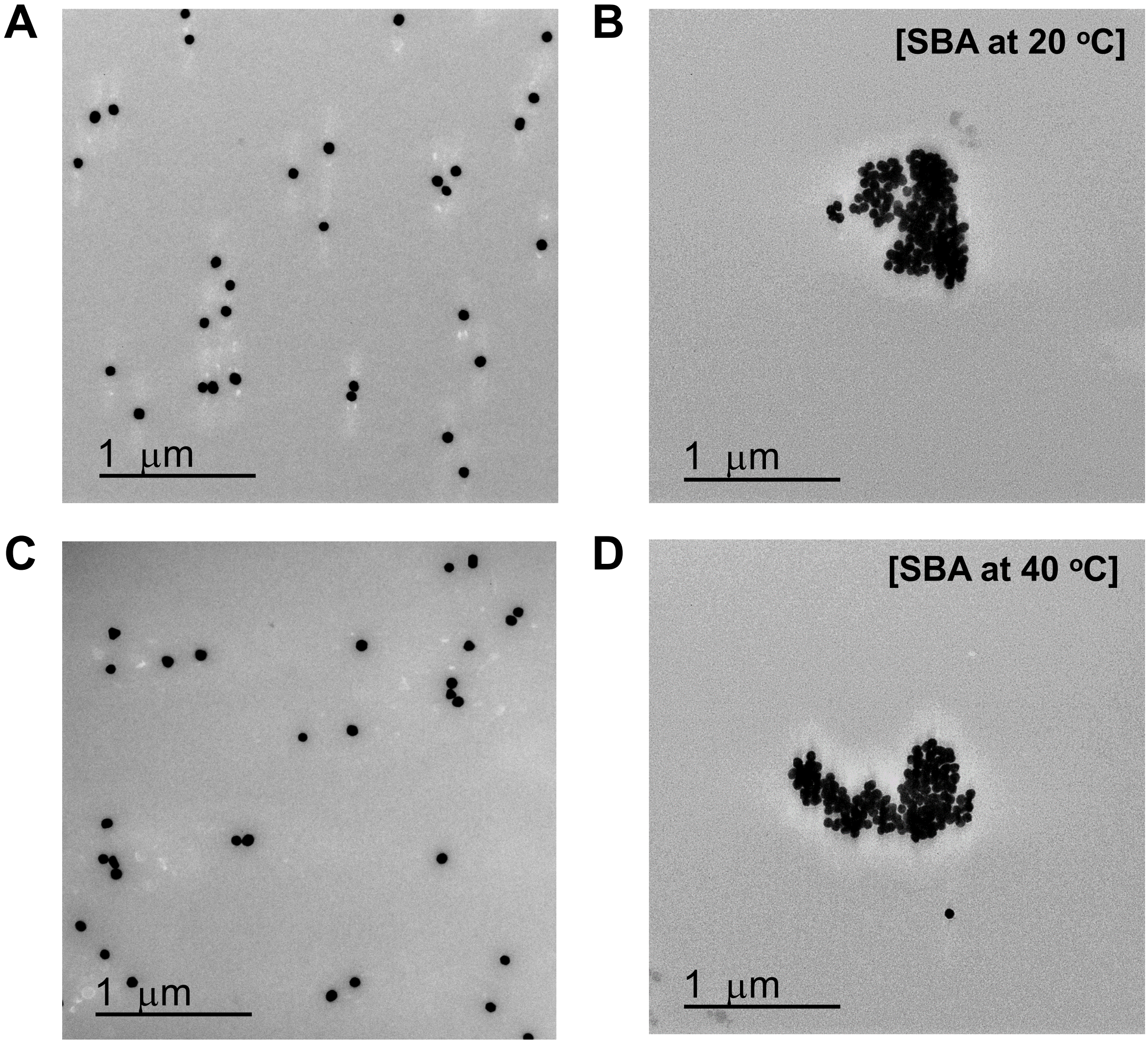

Figure S5. TEM images of pHEA$_{15}$-Gal coated 60 nm gold nanoparticles A), C) non-aggregation without SBA B), D) aggregation with SBA (10 μg mL^{-1}) following 30 minutes of incubation at 20 °C and 40 °C, respectively.

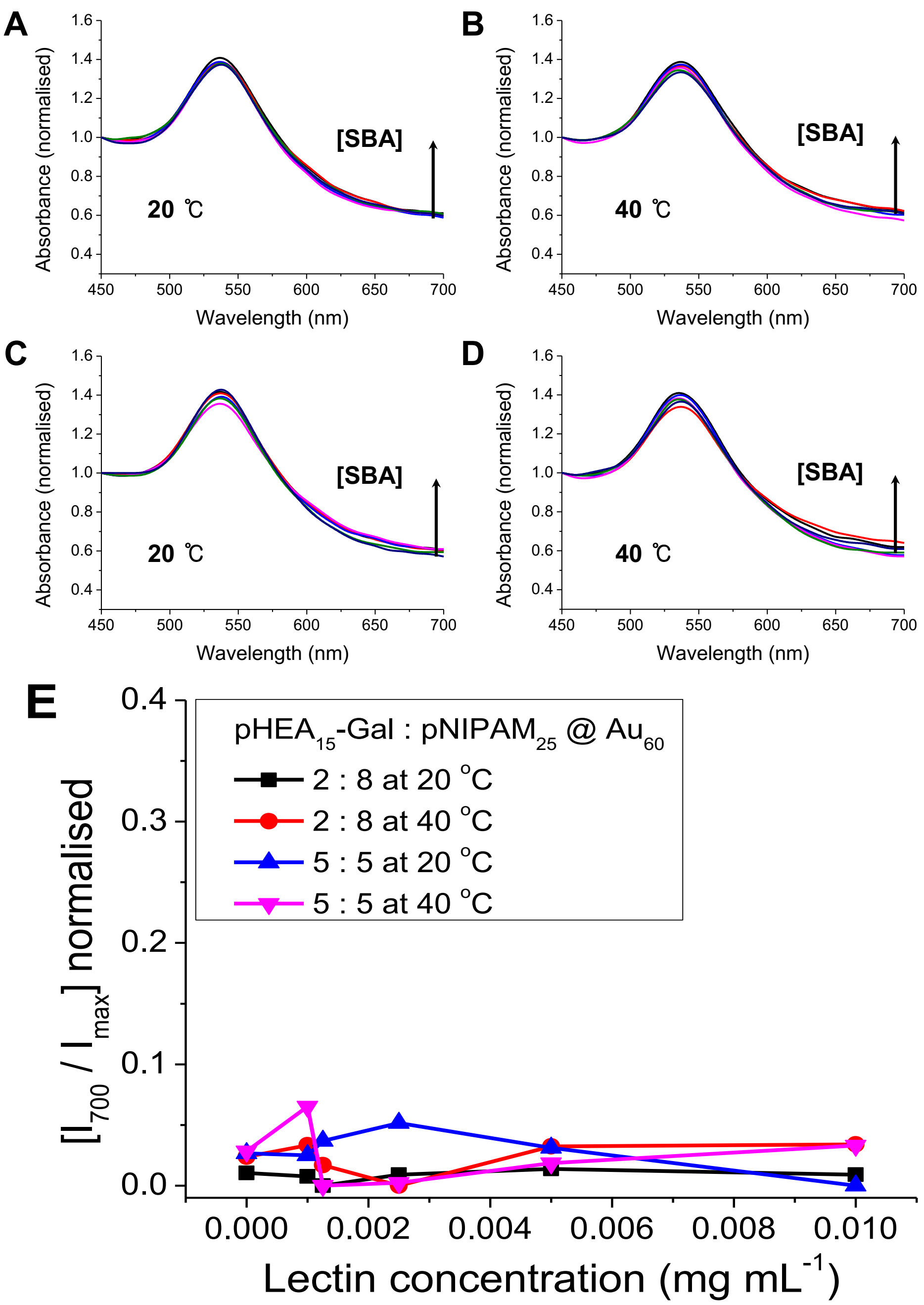

Figure S6. UV-Vis traces of different nanoparticle formulations in presence of serial dilution of SBA (0 – 0.01 mg mL^{-1}) after 30 minutes incubation. Gold particles (60 nm) had [$pHEA_{15}$-Gal]:[$pNIPAM_{25}$] 2:8 at 20 °C A) and 40 °C B); [$pHEA_{15}$-Gal]:[$pNIPAM_{25}$] 5:5 at 20 °C C) and 40 °C D). An increase in Abs_{700} and decrease in Abs_{540} is indicative of binding. All curves normalise so Abs_{450} = 1. E) partial binding isotherm showing relative change in Abs_{700} as a function of concentration to demonstrate affinity of glycoNPs to the SBA.

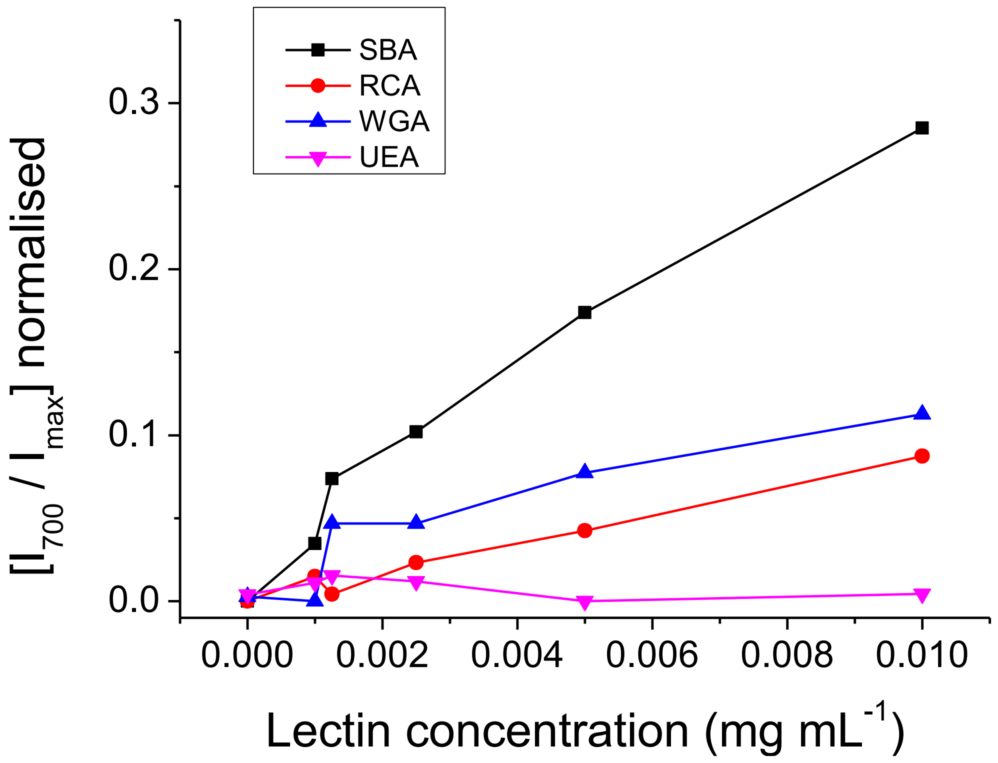

Figure S7. Plot of the ratio of the absorbance intensity at 700 nm and maximum intensity at 540 nm vs. Lectin concentration (mg mL^{-1}) for 1:9 $pNIPAM_{50}/pHEA_{15}$-Gal@Au60 particles after 30mins incubation at 40 ℃.

To test the specificity of the glyco-particle interactions, and rule out non-specific protein binding, control experiments were conducted using BSA as a non-carbohydrate binding protein (Figure S8). Non responsive, galactosylated pHEA coated particles were incubated with a serial dilution of BSA and the UV-Vis spectra recorded, showing no change. Accordingly, the same experiment was undertaken with the pNIPAM containing particles at both low and high temperature (i.e where we see activation towards lectin binding). No change was seen indicating there was no aggregation induced by non-specific effects.

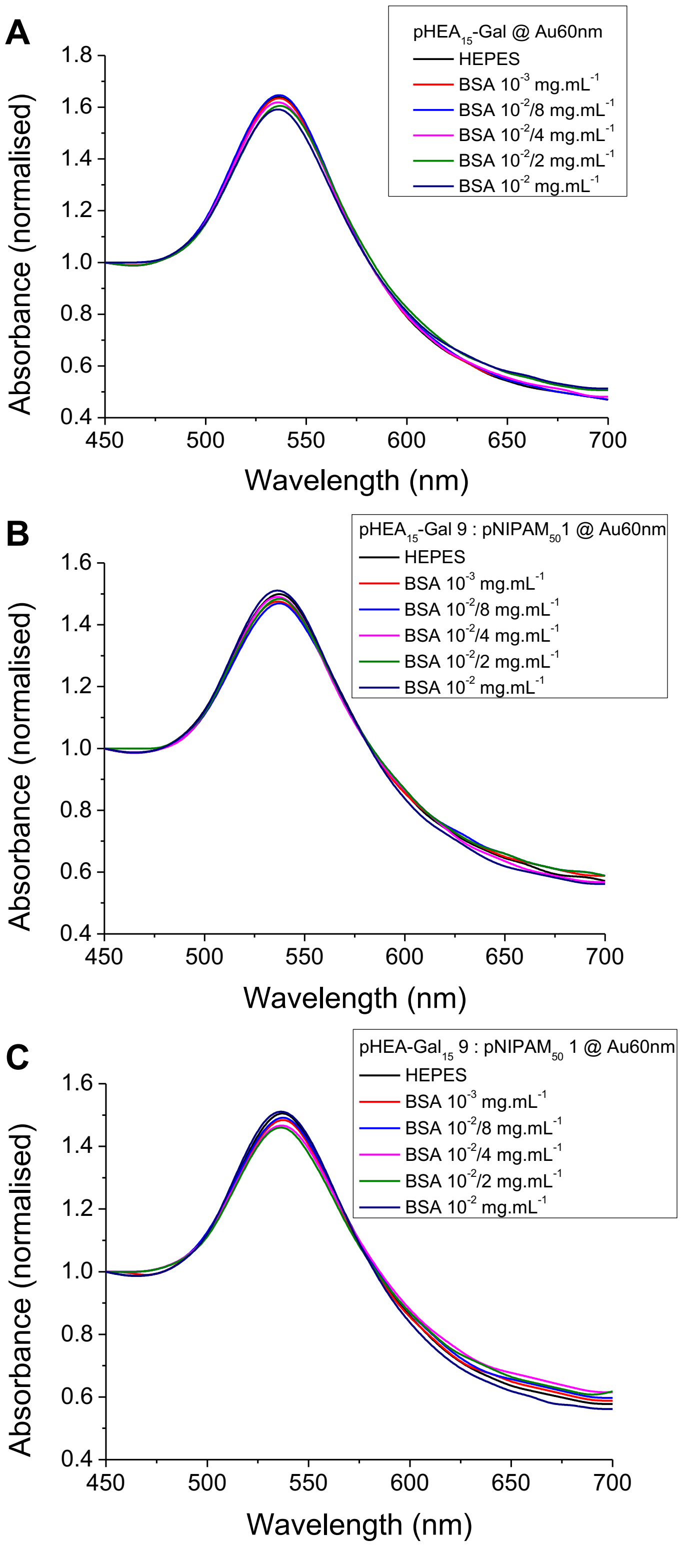

Figure S8. Control experiments for non-specific interactions with non-carbohydrate binding protein, BSA (bovine serum albumin). A) $pHEA_{15}$-Gal@Au_{60} at 20 °C; B) $pHEA_{15}$-Gal (9)-$pNIPAM_{50}$(1) @Au_{60} at 20 °C; C) $pHEA_{15}$-Gal (9)-$pNIPAM_{50}$(1) @Au_{60} at 40 °C

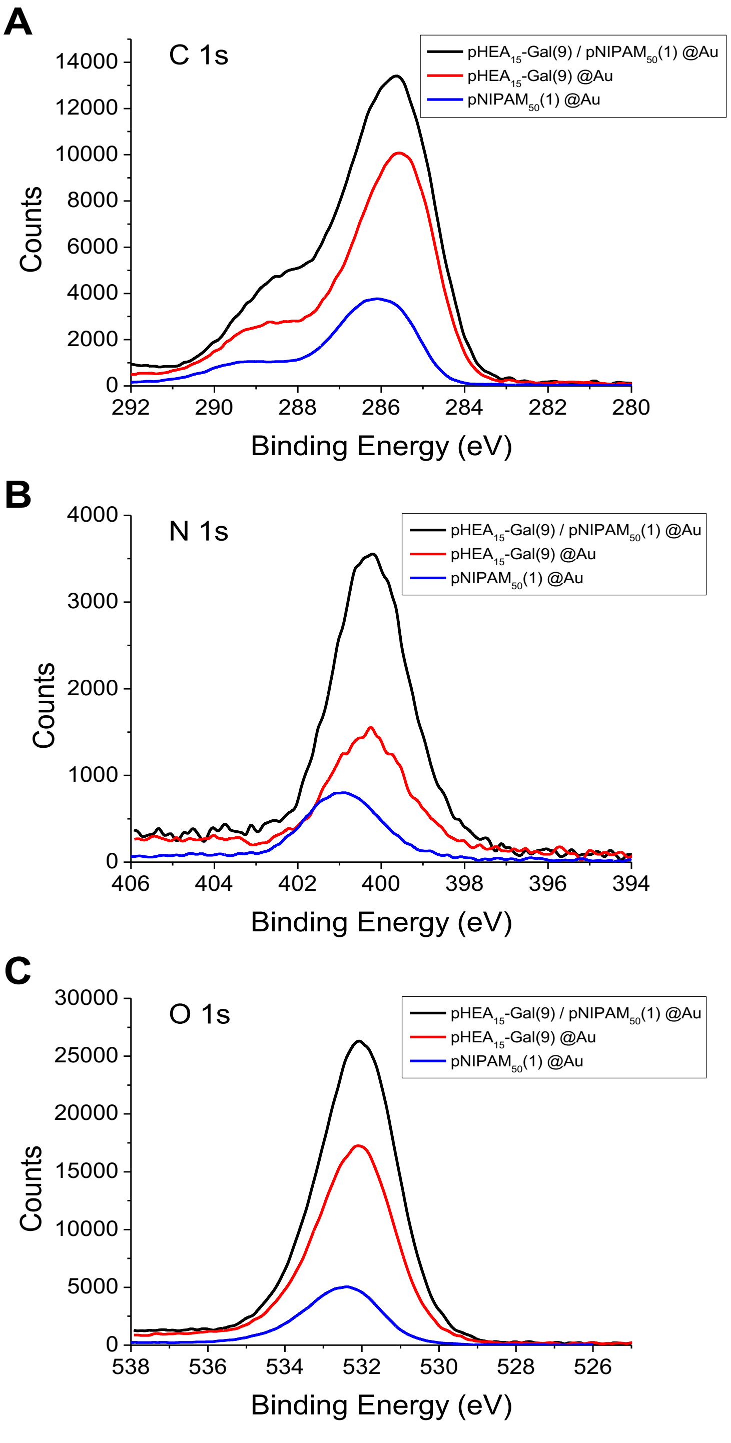

Figure S9. X-ray photoelectron spectroscopy analysis of polymer functionalized gold nanoparticles. Representative high-resolution XPS spectrum of C1s, N1s and O1s region after $pHEA_{15}$-Gal (9)-$pNIPAM_{50}$(1); $pHEA_{15}$-Gal; $pNIPAM_{50}$ conjugation. A) Carbon (C1s) peak, B) nitrogen (N1s) peak and C) Oxygen (O1s) peak from the XPS analysis of polymer/gold hybrid nanoparticles.

Table S2. XPS elemental ratios for Polymer/gold hybrid nanoparticles.

Sample	Au 4f [%]	C 1s [%]	O 1s [%]	N 1s [%]	S 2p [%]
**$pHEA_{15}$-Gal (9) / $pNIPAM_{50}$ (1) @Au_{60}**	0.35	57.76	34.92	6.18	0.79
**$pHEA_{15}$-Gal (9) @Au_{60}**	0.52	60.54	34.37	3.75	0.81
**$pNIPAM_{50}$ (1) @Au_{60}**	1.48	64.37	27.87	5.77	0.50
